# Decision‐Making for Older Patients in Acute Prehospital Situations: A Scoping Review

**DOI:** 10.1111/scs.70148

**Published:** 2025-10-30

**Authors:** Anders Sterner, Bodil Holmberg, Anders Bremer, Anders Svensson, Henrik Andersson, Catharina Frank

**Affiliations:** ^1^ Faculty of Caring Science, Work Life and Social Welfare University of Borås Borås Sweden; ^2^ PreHospen—Centre for Prehospital Research University of Borås Borås Sweden; ^3^ Centre of Interprofessional Collaboration Within Emergency Care (CICE) Linnaeus University Växjö Sweden; ^4^ Department of Nursing Science Sophiahemmet University Stockholm Sweden; ^5^ Faculty of Health and Life Sciences Linnaeus University Växjö Sweden; ^6^ Department of Health Sciences Red Cross University College Stockholm Sweden

**Keywords:** decision‐making, emergency medical services, older patients, prehospital, scoping review

## Abstract

**Background:**

Shared decision‐making aims to ensure that healthcare professionals and patients jointly make decisions regarding the patient's care. However, professionals often find it challenging to implement shared decision‐making with older patients who have cognitive impairments or diminished decision‐making capacity. Research indicates a significant gap in the understanding of how decision‐making processes unfold in prehospital settings.

**Aim:**

The objective of this scoping review was to explore how decision‐making involving older patients in acute prehospital situations is characterized.

**Design and Method:**

This scoping review is based on the Joanna Briggs Institute's guidelines for scoping reviews and is reported using the Preferred Reporting Items for Systematic Reviews and Meta‐Analyses extension for Scoping Review (PRISMA‐ScR).

**Data Sources:**

CINAHL, PubMed, Scopus, PsychINFO and Web of Science were searched to identify relevant studies published between the years 2000 and 2024.

**Results:**

The results are based on 26 studies and indicate that decision‐making among older patients is a conditional process, characterised by collaborative support involving the patient, significant others and healthcare professionals. Barriers to this process include hierarchical dynamics, fear of reprisals and uncertainty regarding the risk–benefit ratio. Factors that support decision‐making include situationally relevant competence, organisational resources and the presence of specific symptoms and signs.

**Conclusion:**

Shared decision‐making with older patients in acute prehospital settings is conditional, often resulting in decisions being made primarily by healthcare professionals. There is considerable room for improvement in how this process is systematically approached. A structured approach is needed—one that assesses the older patient's decision‐making capacity, considers the perspectives of family members, and incorporates input from individuals who know the patient well, all while minimizing hierarchical barriers.

## 1 Background

In the coming decades, it is anticipated that there will be a rapid increase in the number of older people worldwide, particularly in developing countries. It is estimated that the world's population aged 60 and above will double between 2015 and 2050 [1]. In 2023, around two‐thirds of European citizens had retired by the age of 65, and nearly 7% of them retired due to illness or disability [2]. For many, retirement marks the beginning of a period of social connection and self‐fulfilment. This period gradually transitions into increased dependence on others to manage daily life [3]. Therefore, in a European context, the age of 65 can be described as the starting point for the transition into older age.

As a result of demographic changes, an increasing proportion of healthcare resources is being utilised by older people, which has also heightened their need for ambulance care [4], and prehospital care in general [5].

In prehospital settings such as in primary care, ambulance services, and community care, healthcare professionals often face challenges in assessing older patients [6, 7]. This is particularly true for older patients with multiple comorbid conditions, numerous medications, and varying degrees of cognitive impairment [8]. These factors can leave them vulnerable, with functional impairments and cognitive decline, making them weak and frail. Frailty is a strong predictor of falls and mortality, leading to an increased risk of hospital admission. It often indicates a diminished tolerance for exertion, thereby limiting resistance to external stressors related to psychological, social, or environmental obstacles [9]. Such stressors may include loneliness due to loss of community, isolation and loss of meaningful social activities, as well as declining health [10].

When older patients experience acute illness or injury, it is crucial for healthcare professionals to carefully observe and assess their health status. Based on this assessment, they should initiate, implement and evaluate the necessary care interventions [11]. Concurrently, it is essential to consider the patient's experiences, knowledge, beliefs and desires regarding their individual circumstances and needs. This approach can be described as a caring encounter that empowers older patients and enhances their well‐being and health. A caring encounter that fosters trust requires healthcare professionals to be compassionate, genuinely concerned, knowledgeable, skilled and respectful of the older patient both as a person and a patient. Conversely, an uncaring encounter indicates that healthcare professionals are incompetent, indifferent to the older patient as a person, and unconcerned about their impact on the patient. Such encounters may lead older patients to feel distrust, discouragement and a sense of being broken down [12].

Shared decision‐making can be supported through a caring encounter. The concept of shared decision‐making is grounded in patient autonomy, ensuring that the patient is neither abandoned nor deprived of the opportunity to influence their own care [13]. Engaging older patients—through their active involvement in decision‐making and care planning—establishes a more individualised approach to prehospital care, which necessitates shared decision‐making [14]. This way of making decisions refers to a collaborative approach between healthcare professionals and older patients, ensuring that decisions related to the patient's care are made jointly, including formulating the care plan or determining appropriate support interventions [15]. For shared decision‐making to be effective, it is essential that healthcare professionals and older patients exchange all pertinent information. This information must equip all individuals involved with the requisite knowledge to make informed decisions, participate actively and advocate for their own interests [16]. It may involve outlining the available treatment and support options or conveying the advantages and potential risks associated with each alternative. Furthermore, it is assumed that all parties engage in the decision‐making process and reach a consensus on the outcomes, even if this results in a decision to refrain from action [17].

Concurrently, shared decision‐making presupposes consideration of the older patient's cognitive capacity. The information must be tailored to the older patient's specific conditions and requirements; for instance, it may necessitate repetition in both written and oral form or be augmented with visual aids [18]. Shared decision‐making, therefore, centers on cooperation and participation, wherein understanding the other individual's emotions and perceptions is crucial to the decision‐making process. However, shared decision‐making is something more than just methodology. It encompasses both the willingness and the attitude of the involved parties. It is imperative that both healthcare professionals and older patients possess the confidence and readiness needed to engage collaboratively in the decision‐making process [19, 20]. Based on this ideal image of shared decision‐making, there is a significant gap in the understanding of how decision‐making processes unfold for older adults requiring acute care in prehospital settings. A scoping review aimed at identifying research priorities in emergency care revealed 14 key areas, one of which focused on the impact of decision‐making in prehospital emergency care [21]. Therefore, the aim of this study was to explore how decision‐making involving older patients in acute prehospital situations is characterised.

Review questions:

1. What factors influence decision‐making regarding older patients in acute prehospital situations?
2. How is older patients' autonomy or self‐determination regarding decision‐making described in acute prehospital situations?

## 2 Method

This scoping review is based on the Joanna Briggs Institute's (JBI) guidelines for scoping reviews [22], and is reported using the PRISMA‐ScR [23]. The scoping review protocol is not registered online.

### 2.1 Eligibility Criteria

To be included in this scoping review, articles needed to focus on older people (≥ 65) and decision‐making in a prehospital context. Original/primary research papers were included if they were published in peer‐reviewed journals in English between 2000 and 2024. The PCC framework (population, concept and context) guided the construction of eligibility criteria [24].

Population = people on site in situations with older patients with acute care needs.

Concept = descriptions of decision‐making.

Context = prehospital care.

### 2.2 Information Sources

A comprehensive search strategy was developed by the first author and an information specialist at the university library. This strategy was then reviewed and discussed in the research group and agreed upon before being undertaken. The final search strategy can be found in File S1.

The scoping review data search was conducted in April 2023. A complementary search was performed in March 2025. Five databases were selected for their relevance to nursing and healthcare research: Cumulative Index to Nursing and Allied Health Literature (CINAHL), PubMed, Scopus, PsychINFO and Web of Science. All identified articles were collected and uploaded to the reference manager EndNote and duplicates were removed.

### 2.3 Selection of Evidence Sources

**FIGURE 1:**
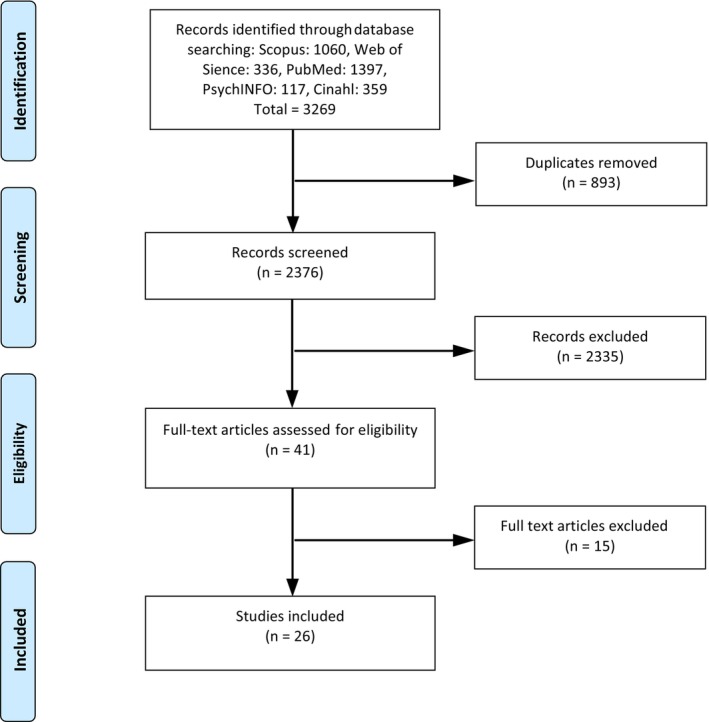
Flowchart of inclusion and exclusion of articles in the scoping review.

A total of 2376 articles were identified and five reviewers, all experienced researchers, independently selected titles and abstracts for full text evaluation, corresponding to the review inclusion criteria, using Rayyan [25]. Reviewer agreement (5/5) generated six articles (*n* = 6). Reviewer disagreements (*n* = 210) were solved as follows: when the article had ≥ 3 reviewers recommending inclusion, the article was included (*n* = 18). When the article had ≥ 3 reviewers recommending exclusion, the article was excluded. When the disagreement was more diverse—for example, one reviewer recommending inclusion, one exclusion, and three being unsure (labelled ‘maybe’ in Rayyan)—this resulted in an independent full text evaluation by the five reviewers (*n* = 17). In total, 41 articles were subject to such an evaluation. Inconsistencies (*n* = 8) were solved by the inclusion of an additional independent reviewer. Finally, 26 articles answering the review questions, regardless of methodological design, were considered eligible for the review. A flowchart of the process is presented in Figure 1.

### 2.4 Data Charting

A data charting form was jointly developed by the research group and full texts of 26 selected articles were reviewed in detail in relation to the inclusion criteria and review questions by three independent reviewers. Each reviewer coded data using a designated colour for each review question. The first and last author then summarised and discussed all codes for each paper in one document. The characteristics of all included articles were abstracted and summarised inspired by JBI's [22] proposed tables and are presented in Table 1.

**TABLE 1:** Summary of the reviewed studies (*n* = 26).

Author, year, country	Objectives	Setting and participants	Method	Results
Amadoru et al. (2018) Australia	To investigate decision‐making around hospital transfer and/or referral of residents to a Residential InReach (RiR) service in north‐eastern metropolitan Melbourne, Australia, from the perspectives of residential aged care facility (RACF) staff, general practitioners (GPs) and RiR registered nurses	Austin health RiR service and RACF RACF staff (31), GP (5), RiR nurses (4)	Qualitative method with individual and group interviews Thematic analysis	RiR utilisation was driven by the following: (i) complexity of decision‐making processes in RACFs; (ii) variability in facility‐based medical and nursing care; and (iii) impact of RiR service outcomes on patients and referrers
Arendts et al. (2010) Australia	To explore the factors that influence the transfer of patients from RACF to hospital emergency departments (ED) and describe features of improved primary care in RACF that could result in reduced transfer	RACF Residents (9), family members (5) of RACF residents, RACF carers (7), RACF nurses (5), ED allied health, medical and nursing staff (7), general practice staff (4)	Qualitative method with group interviews (3) and individual interviews (9) Thematic analysis	Five main themes were identified—staffing and skill mix in RACF, treatment options in RACF, end of life decision‐making, communication and bureaucratic requirements. There was a strong but not universal preference among residents to minimise RACF to ED transfer
Arendts et al. (2015) Australia	To explore perspectives of three groups concerning transfers from aged care facilities to EDs, and also to reveal factors influencing transfer decisions; how active each group was in making decisions; and to what extent groups ceded decision‐making to others.	RACF Residents (11), relatives (14), staff (17)	Qualitative method with individual interviews (42) Content analysis	The three groups substantially differed in their involvement with initiating, and attitudes towards, transfer. Residents were least likely to be involved in the decision, yet most likely to support transfer. Staff felt conflicted between a desire to provide optimal treatment for one ill resident, and their obligations to other residents. Staff perspectives were largely consistent with published data, but new results were found for other informant groups
Bennesved et al. (2023) Sweden	To describe ambulance clinicians' understanding of older patients' self‐determination when the patient's decision‐making ability is impaired	One regional ambulance district Ambulance clinicians (30)	Qualitative design with descriptive phenomenology and dyadic interviews (15) Reflexive thematic analysis	Two themes: (1) movement between explicit and implicit will; and (2) contradictions regarding the patient's best interests. The clinicians’ interpretations are based on an understanding of the patient's situation using substitute decision‐making in emergency situations and conversations that reveal the patient's explicit wishes. Sometimes the clinicians collaborate to validate the patient's implicit will, while at other times they subordinate themselves to others’ opinions. The clinicians find themselves in conflict between personal values and organisational values as they try to protect the patient's self‐determination
Cheek et al. (2005) Australia	To investigate how CPGs support paramedics' care of frail and older adults	Three South Australian aged care organisations providing independent living unit accommodation Older people (31) and family members (10). Stakeholders (GPs, ILU staff, community advisers, ambulance personnel and ED staff) grouped in focus group (14)	Qualitative exploratory and descriptive design Individual in‐depth interviews (41) and focus group interviews (14) Thematic analysis	Eight facets influenced this group of older people's decision‐making with respect to entering the acute care system; they were: expectations of support in the independent living unit not being met; the presence or absence of safety nets; lack of after‐hours support; the desire to remain independent; the GP as pivotal; the influence of others; perceptions of the ED; and having access to information
Cummings et al. (2024) Canada	To develop a conceptual definition of avoidable LTC‐ED transitions and to verify the level of stakeholder agreement with this definition	16 long‐term care (LTC) facilities, 1 ED and 1 EMS in a urban centre in west Canada Participants (80), healthcare aides (20), licensed practical nurses (21), registered nurses (21), LTC managers (10), family members (6), EMS members (9)	Exploratory sequential mixed method. Qualitative semi‐structured individual (25) and focus group interviews (19) Inductive comparison Developing a survey that is then tested quantitative	The definition generated was: A transition of an LTC resident to the ED is considered avoidable if: (a) diagnostic testing, medical assessment, and treatment can be accessed in a timely manner by other means; (b) the reasons for a transfer are unclear and the transition would increase the disorientation, pain, or discomfort of a resident, outweighing any clear benefit of a transfer; and (c) the transition is against the wishes expressed by the resident over time, including through informal and undocumented conversations. There was a high level of agreement with the definition across the four participant groups
Forsgärde et al. (2021) Sweden	To describe extended collaboration in situations when an ambulance was called, as experienced by older patients, a significant other, and ambulance and primary healthcare (PHC) centre personnel	A municipality in the southern part of Sweden Patients (3), significant other (1), ambulance staff (3), staff at the PHC centre (4)	Descriptive qualitative method with a reflective lifeworld research (RLR) Individual interviews (10) Analysed for meanings of the phenomenon	The extended collaboration means that decisions were supported through dialogue by bridging knowledge spaces between person, within‐team and across‐team levels. Through dialogue, experience and knowledge were shared and certainty in decisions was increased. The extended collaboration was built on trust, responsibility taken, shared and entrusted, and the common goal of adapted care for the unique patient
Forsgärde et al. (2023) Sweden	To describe the support for all involved in acute situations when a community health nurse was called, as experienced by older patients, their significant others and health care professionals involved	Three municipalities in southern part of Sweden Patients (3), significant other (1), student nurse (1), GP specialist (1), community health nurses (CHN) (4), ambulance staff (2)	Descriptive qualitative method with reflective lifeworld research (RLR) Individual interviews (12) Analysed for meanings of the phenomenon	Support in decision‐making was received from the knowledge of temporality, which provided a comprehensive understanding based on past and present knowledge of the older patient. The knowledge of temporality allowed for the early detection of new symptoms and facilitated care decisions tailored to the older patient. There was a dependency on preexisting mutual interpersonal support, and confidence developed through relational, caring, and medical competence
Gurung et al. (2022) Australia	To investigate the experience of nurses involved in decision‐making to transfer residents from an RACF to their local hospital ED	Six RACFs located in regional Queensland Aged care nurses (19)	Qualitative method with semi‐structured individual interviews (19) Thematic analysis	Five major themes influenced decision‐making in relation to the transfer of a resident from the RACF to the ED: conflict with key stakeholders; knowledge and experience; policy and process; stakeholder perception; and recognition and support
Halter et al. (2011) UK	To understand the decision‐making processes of emergency ambulance staff with older people who have fallen	London Ambulance Service NHS Paramedics (12)	Qualitative method with individual semi‐structured interviews (12) Thematic analysis	There was a similar assessment and decision‐making process among participants: Prearrival: forming an early opinion from information from the emergency call. Initial contact: assessing the need for any immediate action and establishing a rapport. Continuing assessment: gathering and assimilating medical and social information. Making a conveyance decision: negotiation, referral and professional defence, using professional experience and instinct
Harris et al. (2021) Australia	To investigate how Clinical Practice Guidelines (CPG) support paramedics' care of frail and older adults.	CPGs from nine Australasian paramedic services CPG documents (237)	Summative content analysis for analysing CPG documents (237)	Evidence‐based content relating to older adults was sparse compared to paediatric content. Two overarching decision support domains were identified: patient assessment and management. Inconsistent age descriptors were widespread, particularly in pharmacological guidelines. Five service providers' CPGs contained validated assessment instruments for use with older adults.
Hjalmarsson et al. (2023) Sweden	To illuminate meanings of older people's participation in ambulance care in the presence of municipal care personnel from the perspective of ambulance personnel	One ambulance station in a medium‐sized region in the middle of Sweden Ambulance personnel, RN, RN specialist and emergency medical technicians (11)	Phenomenological hermeneutical method was used to analyse transcripts of narrative individual interviews	The ambulance personnel's lived experience of older people's participation includes passive and active dimensions and involves a balancing act between an exercise of power that impedes participation and equalisation of power that empowers participation. The main theme ‘Balancing dignity in relation to manipulating the body’ included the themes ‘Providing a safe haven’ and ‘Complying with bodily expressions’, which means shouldering responsibility for existential well‐being and being guided by reactions. The main theme ‘Balancing influence in relation to perceived health risks’ included the themes ‘Agreeing on a common perspective’, ‘Directing decision‐making mandate’ and ‘Sharing responsibility for well‐being’, which means shouldering responsibility for health focusing on risks. Influence is conditional and includes performance requirements for both the older person and municipal care personnel
Holmberg B et al. (2023) Sweden	To deductively explore ambulance clinicians' ethical competence when caring for older patients with reduced decision‐ making ability	A southeast region in Sweden with 8 ambulance stations Ambulance clinicians (30), registered nurses (26), emergency medical technicians (4)	Qualitative deductive design within secondary analysis, interviews (15) Deductive content analysis	Ambulance clinicians possess ethical competence in terms of their ethical knowledge, highlighting the need for establishing an interpersonal relationship with older patients. To establish this, they use ethical sensitivity to interpret the patients' needs. Doing this, they are aware of their ethical behaviour, signifying how they must act respectfully and provide the necessary time for listening and interacting
Holmberg B et al. (2024) Sweden	To describe ambulance clinicians' experiences of self‐determination in older patients	Two Swedish regions serving both rural and urban areas Ambulance clinicians (32)	Qualitative and inductively explorative design with focus group interviews (6) Content analysis	The ambulance clinicians assessed the older patients' exercise of self‐determination by engaging in conversation and by being visually alert, to eventually gain an overall picture of their decision‐making capacity. This assessment was used as a platform when informing older patients of their rights, thus promoting their participation in care. Having limited time and narrow guidelines counteracted ambulance clinicians' ambitions to support older patients' general desire to avoid hospitalisation, which resulted in an urge to displace their responsibility to external decision‐makers
Holmberg M et al. (2024) USA	To explore the attitudes and perceptions of paramedics in a US context regarding self‐determination in elderly patients who need emergency care provided by EMS	Expert panel of US paramedics (21) in Washington State	Exploratory design, and data were collected and analysed using a Delphi technique	A total of 15 experts completed all three rounds, leaving a total response rate of 71%. Finally, 87 out of 108 items reached consensus, of which 60 were ‘agree’ and 27 were ‘disagree.’ The paramedic–patient relationship is a core part of assessing and handling ethical challenges within an advanced practice influenced by the paramedics' educational level and/or the patient's physical/mental status. Within a ‘find it fix it’ modus operandi, there is a need to increase paramedics’ competence in understanding and handling advanced ethical challenges in relation to ethical values such as autonomy and self‐determination in older patients
Kihlgren et al. (2003) Sweden	To address two research questions: (1) What factors and aspects influenced community RNs' decisions in referrals of older patients for emergency treatment? (2) What kind of support may be required to facilitate this decision‐making?	Home Care Services (HCS) in five communities and primary health care centres in four communities Registered nurses (10) from HCS and consultant physicians (4) from primary health care centres	Qualitative study with individual interviews (14) Content analysis	The categories were: own competence, knowledge about the patient, and a supportive working environment. The main theme was ‘To feel safe in one's role—a basis for decision‐making’. High demands were put on the nurses’ competence and their burden of responsibility became too great. This influenced decision‐making negatively, if nurses felt that they were lacking in their own personal competence. Training in documentation for the nurses was required, as well as the need for organisations to provide staff with sufficient time for accurate documentation. A greater input of nursing and medical care was required to make it possible for patients to be cared for at home if they so wished
Neiroff et al. (2019) Canada	To estimate the prevalence of, and adherence to, ‘no transfer to hospital’ advance directives (ADs) in long‐term care (LTC) and to explore the circumstances leading to transfers against previously expressed directives	Ten nursing homes in Nova Scotia Residents' charts and emergency health service database (748)	Mixed method approach LTC chart and health services from participants Pearson chi‐square test, Shapiro–Wilk normality test, Mann Whiney *U*, Kruskal Wallis, and content analysis	Paramedics were called for 80.5% of residents and 73.6% were transferred to hospital, 51.3% of whom had explicit ADs to the contrary. The majority of those were transferred for fall‐related injuries, followed by medical illness. Unclear care plans, symptom control, and perceived need for investigations and procedures all influenced transfer decisions
Nicholson et al. (2022) UK	To explore the factors that influence paramedic decision‐making when considering whether to convey an adult aged 65 years and over with a minor head injury to the ED	South Western Ambulance Service in Southwest England Paramedics (10) and ambulance service data	Multiple method: retrospective analysis of ambulance service data and semi‐structured telephone interviews (10) Thematic analysis	South Western Ambulance Service NHS Foundation Trust (SWASFT) attended 15,650 emergency calls to patients aged 65 and over with minor HI, with 70.5% conveyed to ED. 81% of conveyed patients met NICE and JRCALC guideline criteria for conveyance, with the remainder conveyed due to wound care or other medical concerns. The framework developed from the interviews comprised four themes: resources, patient factors, consequences and paramedic factors
Simpson et al. (2017) Australia	To explore the decision‐making process used by paramedics when caring for older fallers	South Western Ambulance Service Paramedics (33)	Qualitative, grounded theory (GT) study; data were collected from face‐to‐face interviews (13), with focus groups (4) introduced during the theoretical sampling phase to help clarify emerging theoretical concepts Analysis commenced after the first interview and continued concurrent to the ongoing data collection process	When caring for older fallers, paramedic decision‐making is profoundly affected by ‘role perception’, in which the individual paramedic's perception of what the role of a paramedic is determines the nature of the decision‐making process. Transport decisions are heavily influenced by a sense of ‘personal protection’, or their confidence in the ambulance service supporting their decisions. ‘Education and training’ impacts on decision making capacity, and the nature of that training subliminally contributes to role perception. Role perception influences the sense of legitimacy a paramedic attaches to cases involving older fallers, impacting on patient assessment routines and the quality of subsequent decisions
Sunner et al. (2022) Australia	To explore whether an intervention using visual telehealth improves care outcomes for residents in RACFs during acute illness events from the perspective of nurses from RACFs and EDs	Four EDs and 16 RACFs, Local Health District (LHD) in New South Wales ED nurses (6), RACF nurses (19)	Qualitative design with interpretive descriptive design and focus group interviews (6) Analysed with interpretive descriptive method	There were four overarching themes that emerged from the six focus groups; facilitated person centred care; built confidence, relationships and trust; enabled bidirectional communication that strengthens decision making, but there were issues with technology access, connectivity and usability between the acute care setting and the RACF
Svensson et al. (2021) Sweden	To empirically explore attitudes among Swedish ambulance clinicians (ACs) regarding older patients' self‐determination in cases where patients have impaired decision‐making ability, and who are in urgent need of care	Ambulance stations in two regions, southern Sweden Prehospital emergency nurses (31), registered nurses (10), emergency medical technicians (7)	Explorative design using a modified Delphi technique comprising four rounds	Round 1 identified 108 items divided into four categories: (1) attitudes regarding the patient, (2) attitudes regarding the patient relationship, (3) attitudes regarding oneself and one's colleagues, and (4) attitudes regarding other involved factors. After four rounds, 72 items (62%) reached consensus
Tate et al. (2020) Canada	To describe the perspectives of health care aides (HCAs) regarding their roles and communication processes in clinical decision making about resident health leading to decisions‐to‐transfer in ambiguous situations	Five nursing homes in a major urban area in Western Canada Health care aides (HCA) personnel (20)	Qualitative‐focused ethnographic study with individual interviews (6), five joint interviews (*n* = 2), and one focus group (*n* = 4) Analysis with codes and categories	Hierarchical reporting structures influenced HCAs' perceptions of nurse responsiveness to their concerns about resident condition, which influenced communications related to transfers. Communication processes in long‐term care (LTC) facilities and the value placed on HCA concerns are inconsistent
Vicente et al. (2013) Sweden	To describe patients' lived experience of participating in the choice of healthcare when being offered an alternative care pathway by the EMS, when the individual patient's medical needs made this choice possible	Geriatric ward and community acute care centre Older patients (9)	Phenomenological reflective life‐world research (RLR) approach with individual semi‐structured interviews (9) Phenomenological analysis	Essence of the phenomenon is described as ‘There was a ray of hope about a caring encounter and about being treated like a unique human being’. Five meaningful constituents were identified: endurable waiting, speedy transference, a concerned encounter, trust in competence, and a choice based on memories of suffering from care
Vilke et al. (2002) USA	To obtain medical follow‐up and determine reasons why elderly patients access paramedics via 9–1‐1 and then refuse transport	EMS in San Diego, California Older patients (121)	Telephone follow‐up survey (121) Descriptive statistical analysis	Financial concerns were a major determinant in refusing to be transported. Overall, 70% of the patients reported receiving follow‐up medical care. Care was obtained at an ED via a second 9‐1‐1 caLL in 16% of cases, at an ED via private vehicle in 13%, at an urgent care clinic by a private vehicle in 35%, and with a family physician via private car in 38% of cases. Of the patients who obtained follow‐up, there was a 32% hospital admission rate, with 39% of those admitted to an intensive care unit setting
Voss et al. (2020) UK	To investigate the factors that influence the decision‐making process of paramedics during calls to older people with dementia, with a view to providing paramedics with adequate support to enhance care in this patient group	One region of a single United Kingdom ambulance service Paramedics (16)	Qualitative study with phenomenological approach. Observation, individual interviews (16) Observation assessment input from family members and actions taken by the paramedic as field notes and interviews. Document and thematic analysis	Four main themes emerged from the data concerning the way that paramedics make conveyance decisions when called to people with dementia: (1) Physical condition: The key factor influencing paramedics' decision‐making was the physical condition of the patient. (2) Cognitive capacity: most of the participants preferred not to remove patients with a diagnosis of dementia from surroundings familiar to them, unless they deemed it essential. (3) Patient circumstances: this included the patient's medical history and the support available to them. (4) Professional influences: participants also drew on other perspectives, such as advice from colleagues or information from the patient's GP, to inform their decision‐making
Watkins et al. (2024) Australia	To explore paramedics' experiences and perspectives about attending and managing older adults who had fallen	Paramedics and ambulance officers (14) with at least 1 year of clinical experience in Western Australia	Qualitative, exploratory study with individual semi‐structured interviews Inductive thematic analysis	The main theme identified that experiences were positive when attending patients with high‐acuity medical problems or injuries following falls because binary decision‐making (transport vs. non‐transport) was appropriate. Themes highlighted that decision‐making for low‐acuity falls attendances was a complex balance between (1) patient context, (2) risk management, (3) paramedic reactions, and (4) the lack of alternate referral pathways available. Experiences could be stressful and frustrating when attending falls call‐outs for older adults with no injuries or medical problems. Participants concurred that when transport to hospital was not required there were no available alternative pathways to refer onwards for appropriate health or social care

### 2.5 Data Analysis

**FIGURE 2:**
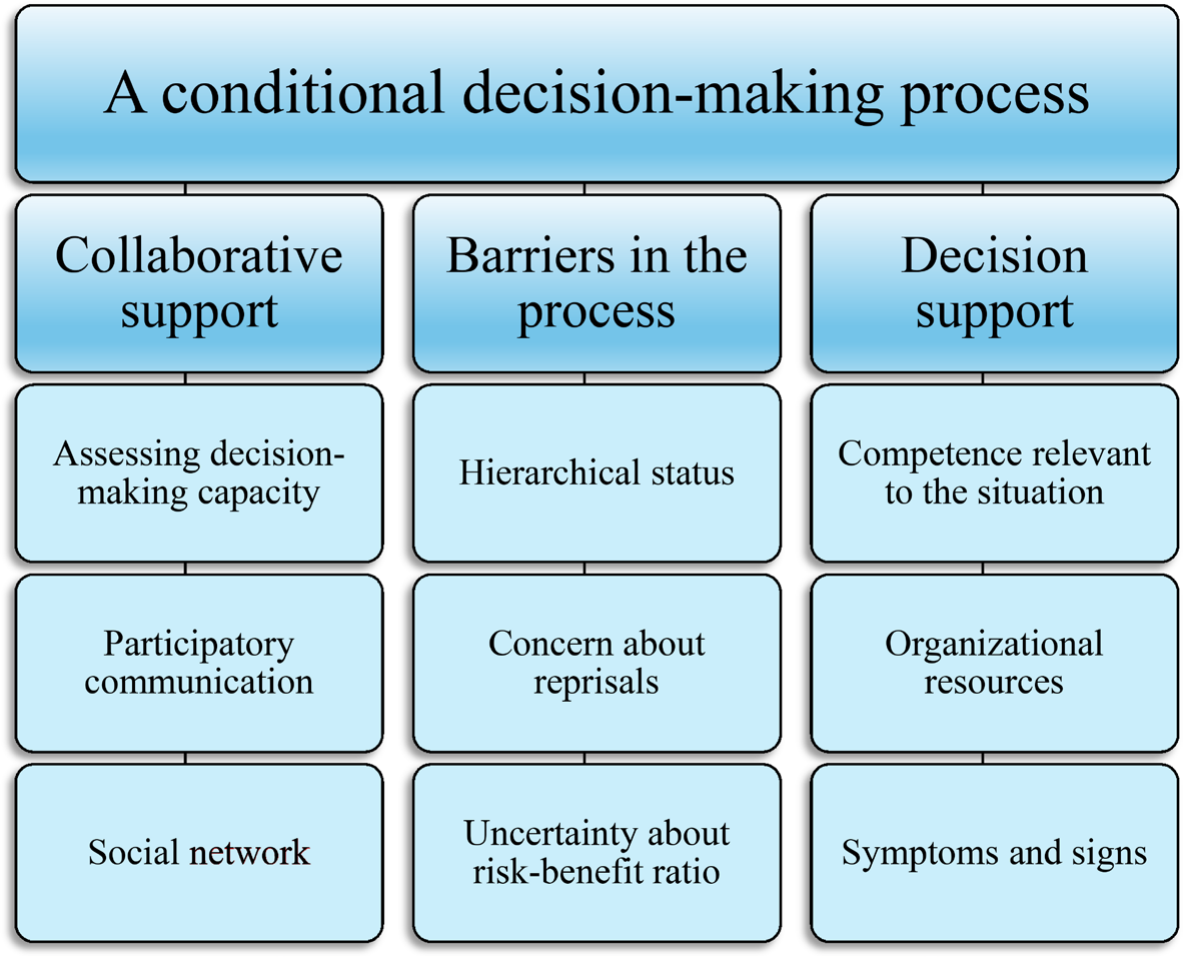
Description of theme, generic categories and subcategories.

As recommended by JBI, a basic content analysis using Elo and Kyngäs' [26] framework for inductive extraction and analysis was applied. Data extracted from each article were read and reread by the first and last author to gain familiarity with the content. Subsequently, the two authors coded the data independently. These codes were summarised in a coding sheet and then discussed to create initial categories. These initial categories were discussed in the research group, leading to the creation of a theme, generic categories and subcategories (Figure 2).

### 2.6 Ethical Considerations

In accordance with ethical research guidelines and principles, all articles included in this review have either been granted ethical approval or include a discussion explaining why ethical approval was not necessary according to national laws and regulations.

## 3 Results

### 3.1 Theme: A Conditional Decision‐Making Process

#### 3.1.1 Generic Category: Collaborative Support

##### 3.1.1.1 Subcategory: Assessing Decision‐Making Capacity

The older patient's capacity for self‐determination was assessed by healthcare professionals in a collaborative process with the older patient [27, 28, 29, 30, 31, 32, 33, 34]. The assessment takes place spontaneously and is initiated by professionals, depending on their confidence to act independently in relation to the patient and their family. These healthcare professionals have prior situational experience, along with self‐confidence [28, 35] and a solid understanding of the patient's situation and capacity for self‐determination [28, 29, 31, 35, 36, 37]. Healthcare professionals assessed the self‐determination of the older patients and furthermore interpreted the situation concerning cognitive ability, communication ability, degree of mobility, body language, surrounding environment, care documentation and access to support from those who best know the patient and their interests [28, 29, 30, 31, 33, 34, 38, 39], and the older patient's ability to understand instructions and prompts [40].

##### 3.1.1.2 Subcategory: Participatory Communication

The decision‐making process is influenced by participatory communication, which is manifested in a respectful mutual interaction between people involved in the situation [27, 33, 36, 38, 39, 40, 41, 42, 43]. Mutual interaction means that people involved in the situation listen to each other to jointly optimise the development of a balanced decision for or with the older patient. Healthcare professionals, significant others, and the older patient are all involved but are not always in agreement. However, everyone has the space to make their voice heard [27]. Healthcare professionals' intentions are primarily to listen to the explicit and implicit wishes of the older patient and the family [28, 34, 36, 44] in a dialogue requiring both time and space [27, 31, 33, 36] explaining the available choices and their consequences. Health professionals also consider the use of language and the time spent on the care relationship. A supporting dialogue with available people is a requirement for the evaluation of the patient's capacity to exercise their self‐determination [28, 29, 31, 33, 36].

Involvement in decision‐making depends on the extent to which the older patient, family members, and healthcare professionals have knowledge and are prepared for acute events, how familiar they are with the patient's relevant medical history, and how aware they are of their right to be involved in the situation [27, 28, 32, 35, 40, 41, 42, 44, 45, 46]. Even if people involved in the decision are not in agreement, arriving at a decision is easier when the older patient is involved in the decision‐making process; although it may be the case that the older patient and family either hand over or are perceived to hand over decision‐making to someone else [29, 36, 41, 43, 46, 47].

##### 3.1.1.3 Subcategory: Social Network

An older patient's social network supports the decision‐making process in acute situations. Social networks involve partners, families and friends. The support can include discussions about the acute situation that has arisen and the uncertainty regarding when and whether to call for help [27, 46, 48]. Being alone without a social network present makes the older patient feel insecure and can result in calling for help earlier [40]. In the joint discussion, advice and other people's experiences are shared regarding how the older patient and family think about the situation. Reasoning with others is significant for the older patient but also affects the healthcare professionals who arrive at the scene. Significant others can confirm the acute event and describe the older patient's functioning under normal circumstances. Healthcare professionals’ awareness that an older patient is supported by a social network can influence clinical decision‐making [28, 30, 34, 39, 40]. However, when the family lacks the means or capacity to provide support, healthcare professionals may face increased pressure to make decisions that align with the family's wishes [34, 36].

#### 3.1.2 Generic Category: Barriers in the Process

##### 3.1.2.1 Subcategory: Hierarchical Status

The decision‐making process in acute situations is affected by hierarchical structures and is dependent on the status of healthcare professionals. This means that assessments are valued differently and can be questioned by other healthcare professionals [28, 29, 32, 35, 49]. Some healthcare professionals who are called to the older patient's home have high levels of medical competence and are highly valued. Their words carry weight and are influential in the decision‐making process. The older patient is greatly influenced by their advice and recommendations and tends to let the healthcare professionals decide completely on their behalf as the patient [31, 32, 43].

Healthcare professionals who work closely with older persons generally have lower levels of education and are graded as being at the bottom of the hierarchical structure [32, 49]. These professionals can have difficulty making their voices heard and are rarely involved in the decision‐making process [49]. Furthermore, hierarchical structures affect how different groups of healthcare professionals listen to each other, and this accordingly affects the trust they have in each other's competence to assess the situation and follow the wishes of the patient [29, 32].

##### 3.1.2.2 Subcategory: Concerns About Reprisals

Healthcare professionals' concerns about reprisals can affect the decision‐making process in emergency situations. Decisions are sometimes influenced by healthcare professionals' fear of consequences rather than by the older patient's primary needs [28, 29, 30, 35, 37, 38, 39, 40, 41, 44, 49]. Healthcare professionals choose different strategies to avoid possible consequences and thereby protect themselves. In the acute situation, professionals act based on assumptions about what might happen in the long term, driven by the fear of being reported afterwards, ending up in litigation, being questioned by colleagues, or receiving complaints from the patient, their family, and/or related organisations [29, 35, 37, 38]. Strategies employed include manipulation [41], such as persuasion and diversion [28, 29, 31, 39] of the older patients and their family members when they express a will that is not in line with the healthcare professionals’ will [36]. Persuasion takes place both directly in connection to the older patient and via family members and other professionals on‐site. An additional strategy is to ask the older patient to sign a document declining transport to the hospital [39]. In this situation, healthcare professionals act mainly to protect themselves from possible consequences.

##### 3.1.2.3 Subcategory: Uncertainty About Risk–Benefit Ratios

The decision‐making process in acute situations is affected by uncertainty in assessments regarding the risk–benefit ratio for the older person's health. Both healthcare professionals and the older patient, along with family members, balance the risks and benefits of going to hospital or staying at home [27, 30, 33, 38, 39, 40, 41, 46, 48, 50]. Healthcare professionals’ fear of consequences is sometimes based on uncertainty about what is best for the older patient in need of care. The decision is mainly influenced by the patient's needs. The uncertainty is usually around the risks and benefits they may mean for the patient, especially in situations where the decision is made to stay at home rather than be transported to a hospital [27, 35, 39, 40, 41, 44]. For older patients, this decision means a trade‐off, where they use different options before contacting help because it is highly valued to be able to stay in their own home and be offered care there [40, 46, 48]. In addition, there is sometimes a fear of becoming dependent if emergency assistance involves hospitalisation [46].

#### 3.1.3 Generic Category: Decision Support

##### 3.1.3.1 Subcategory: Competence Relevant to the Situation

The decision‐making process is influenced by the level of competence within the organisation [28, 35, 38, 40, 44, 45, 48]. Healthcare professionals need to be competent in making decisions, as these are complex situations and based on both objective signs and subjective symptoms of older patients [43, 48]. Competence is dependent on the healthcare professional's education, including educational content and level of education [30, 32, 35, 37, 40, 44, 45, 49]. Another relevant aspect of competence is personal knowledge concerning the patient's habitual situation, which is especially important in detecting subtle signs [29, 33, 42, 49] and thereby streamlining the decision‐making process [27]. Lack of relevant competence increases the number of decisions made to transport older patients to care facilities [29, 40, 45].

##### 3.1.3.2 Subcategory: Organisational Resources

Organisational resources such as availability and routines are supportive in decision‐making. Resources consist of the number of healthcare professionals available, the number of hospital beds in the local hospital, distance to hospital, technical innovations [29, 30, 34, 35, 41] and access to care and higher medical competence within a reasonable time, especially after office hours [32, 38, 44, 46]. Lack of resources or absence of resources results in sub‐optimal solutions [41]. One way to get around a lack of resources is the ability to contact individuals with higher levels of medical expertise [38, 47].

Documentation is supportive in decision‐making [35, 38, 44] and includes various types of notes and advanced care plans or advanced health directives [32, 38, 44]. Care plans provide support by stating the older person's values, life goals and desired outcomes or direction of care and treatment [32]. To be supportive, it is important that the documentation is well‐written, not too extensive, easily accessible and up to date [28, 32, 35, 38].

Assessment instruments are supportive in decision‐making; for example when the instrument includes a systematic review of the older patient's symptoms and signs. Sometimes there are no instruments for the specific situation, forcing ad hoc decision‐making [27, 30], or the instruments lack clear definitions and can be sweeping in their descriptions of which patients the specific assessment instrument should be applied to [51]. The application of assessment instruments and clear guidelines is regulatory and helps guide decision‐making but they come at the expense of healthcare professionals’ room for manoeuvre, which could be perceived negatively, especially if judgements about what is best in the situation for the older patient differ from the guidelines or outcomes from instruments [28, 29, 30, 31, 40, 44]. One form of support in decision‐making in these situations can be to contact higher medical expertise.

##### 3.1.3.3 Subcategory: Symptoms and Signs

The overall picture of risk factors, symptoms, and signs of acute illness or injury aids in the decision‐making process. Some diagnoses, conditions, or drug intakes are red flags and indicate a need for transport to hospital without discussion [28, 29, 31, 37, 38, 39, 40, 43, 44, 49]. However, some of these indications may also lead to avoiding transport to hospital [29, 34]. Also, the level of consciousness, pain, injuries from falls, repeated falls or long times spent on the floor indicate immediate transport to hospital [30, 31, 39]. Healthcare professionals are aware that severe illness can exist without abnormal signs and can cause rapid changes to the patient's condition [27]. Symptoms and signs may also indicate the need for further decision‐making support through examination or treatment by higher medical expertise available elsewhere [32, 35, 38, 41, 45, 46, 52], or other socially orientated interventions or measures [31, 51], which are not in line with the patient's own wishes [36].

## 4 Discussion

As shown in the results, the promotion of older patients' participation in decision‐making in acute prehospital situations appears to be a goal for all parties involved. However, this goal can be difficult to achieve, as the result of the older patient's participation in the decision‐making process is repeatedly conditioned and often interrupted and hampered by social and organisational aspects. This may lead to decisions being made by healthcare professionals who have the highest level of medical knowledge, rather than by healthcare professionals with lower levels of education who work closely with older patients in their daily lives. Consequently, regardless of who the healthcare professional is, shared decision‐making seems to revolve around what Sandman and Munthe [13] would call paternalism based on a joint rational dialogue with the patient. However, this type of paternalism seems to be complicated by power relationships between professionals.

The decision‐making process is influenced by participatory collaboration, which is manifested in a mutual interaction between people involved in the situation. Even though the involvement of the older patient and their family members depends on their preparedness for acute events, their familiarity with the relevant medical history, and their awareness regarding their right to be involved, the impact of knowledge from healthcare professionals well known to the patient may also need to be more highly valued in acute situations. In alignment with this, a study exploring the perspectives of older patients and family members highlights the importance of a personalised approach to decision‐making. Some wish to be actively involved and well informed, and they express dissatisfaction when the decision‐making process feels like a ‘one‐directional’ decision imposed by healthcare professionals. While such top‐down decisions may be accepted in acute situations that allow little time for reflection, older patients and their families often feel that events unfold without their full awareness, which can lead to frustration. These findings align with results from another study conducted in homecare settings. In that study, nurses described how they developed in‐depth knowledge of older patients over time—not only regarding their physical limitations but also in terms of their broader life context, including identity and personal life stories. This comprehensive understanding enables healthcare workers to detect subtle physical and mental changes in older patients, such as signs of existential distress. Such insights are made possible through regular home visits, which foster a caring relationship grounded in mutual trust and openness. As a result, older patients may experience a sense of security, even during acute situations when their sense of control is minimal [53].

The results show that older patients have a voice in decision‐making, even if the decision depends on the competence healthcare professionals have in caring for the older patient. However, the treatment of patients is not always carried out in a caring manner that respects the dignity and autonomy of the older patient. Such a violation of dignity may disrupt the older patient's feeling of being a whole person and thus cause suffering. When this suffering emanates from the way healthcare professionals treat the older patient, it may be labelled as ‘care‐suffering’. This is a kind of suffering that should not exist at all, as it is caused by inadequate care which, to some extent, is a failure to see and judge what older patients really need [54, 55]. Furthermore, when older patients perceive a lack of self‐determination, they may experience their dignity as being threatened [56].

Furthermore, the results show that older patients' decision‐making is conditioned when they lack cognitive or communicative ability, where healthcare professionals turn to family members who are supposed to know the older patients well. However, not all older patients and family members are alike. One study showed that, regardless of how decision‐making was conducted by healthcare professionals, some patients and family members accepted strict paternalism in acute situations that provide little time for reflection, while others wished to be involved, to be kept well‐informed, and disliked experiencing decision‐making as a ‘one‐directional’ route led by healthcare professionals [57]. A key prerequisite for effective participation in decision‐making is receiving adequate information proactively, without needing to ask for it. When information is lacking, it can lead to confusion and hinder meaningful involvement [57]. This underlines the moral necessity of taking time to inform older patients properly, in line with the ideals of shared decision‐making. However, providing a lot of information in an acute situation may also risk overloading older patients with information in a way that blocks their thinking, confuses them and forces them to involuntarily transfer decision‐making to others. These risks make decision‐making more complex, as other persons may differ in values, power, and the ability to interpret given information [58]. This speaks to Sandman and Munthe's assertion that models of shared decision‐making cannot be reduced to either paternalism or patient choice [13]. Ideally, the ‘shared rational deliberative joint decision’ model should be adopted, but if the patient and professional fail to reach consensus, there is reason to pursue the ‘professionally driven best interest compromise’ model, as this model harmonises different values at stake (patient best interest, patient autonomy, patient adherence and a continued care relationship). However, in acute prehospital situations, it is important to emphasise the need for a flexible and dynamic application of different decision‐making models.

The results that is, of the present study reveal the conditioning impact of hierarchical structures in shared decision‐making, where suggestions made by healthcare professionals with high levels of medical knowledge have a high impact, while healthcare professionals who work closely with the patient tend to be overlooked and rarely listened to. Such an approach may be valid in situations with ‘red flag’ diagnoses, where the older patient is willing to transfer their decision‐making to those who are assumed to have the highest level of medical competence. However, the results also elucidate that there is a need for decision support when it comes to detecting subtle signs that can be best discerned by someone who has personal knowledge of the older patient. Such knowledge is described as important, as it may shorten the decision‐making process.

As the results show, older patients are supposed to be at the top of the hierarchical structure. Healthcare professionals' primary ambition is to listen to the wishes of older persons to facilitate the decision‐making process. If needed, they also involve family members in gathering crucial information. However, there are situations in which the participation of older patients is conditioned and limited without a clearly justifiable reason. Factors such as healthcare professionals' lack of competence, fear of repercussions, or efforts to balance perceived risks and benefits may lead them to steer patients into accepting their own recommendations. These factors often result in a decision to transport the older person to a care facility. However, such decisions may inadvertently undermine the older patient's sense of autonomy, particularly when hospitalisation is associated with a fear of becoming dependent. From an ethical standpoint, persuasion can sometimes support older patients in reconsidering and articulating their preferences in a more feasible manner. Yet, it also carries the risk of fostering a paternalistic approach—especially when persuasion is based on information or trust that the patient or their family has placed in healthcare professionals. In such cases, the boundary between guidance and coercion may become blurred, potentially compromising the patient's autonomy [58]. Simultaneously, there may be acute situations where healthcare professionals can use paternalism‐by‐persuasion in a beneficial way; that is, when they use their knowledge about the patient in combination with their greater insight and medical knowledge to look forward in time and predict possible outcomes in ways that the patient themselves cannot. Such a strategy can even be described as respecting the patient's autonomy, as the patient may later agree to the decision when the acute situation has passed, and they can think more rationally [59]. This underlines the need for healthcare professionals to promote an authentic encounter that implies a compassionate and open attitude, combined with sensibility, when interpreting older patients’ needs. To achieve this requires healthcare professionals to harbour genuine feelings of goodwill and a holistic view of caring, as well as an ability to be physically and emotionally attentive in situations and thus present in the dialogue in both listening and responding. An authentic encounter requires the courage to fully become involved in the older patient's world [60]. Therefore, a patient‐centred approach should extend beyond the practice of shared decision‐making to also encompass a lifeworld perspective—one that empathetically considers how older patients perceive and experience their world. This includes efforts to understand how their personal life stories shape their current situation; how they interpret and find meaning in their surrounding world in light of physical limitations; and how their emotional state influences their relationships, self‐perception and outlook on the future. When healthcare professionals succeed in engaging with older patients from a lifeworld perspective, they can offer more than just technical solutions—they can also help identify meaningful paths forward in both care and life [61]. Such an approach aligns with the principles of a caring encounter, which has the potential to empower older patients and enhance their overall well‐being and health [12]. Based on this study's aim to explore how decision‐making involving older patients is characterised in acute prehospital situations, our findings highlight the importance of healthcare professionals at all hierarchical levels working to remove barriers to participation. This involves not only gathering medical information, but also actively incorporating lifeworld‐related insights as a foundation for shared decision‐making—particularly in situations where patient involvement is challenging.

## 5 Limitations

There are some limitations in this scoping review which merit consideration. All the studies included in this review originate from high‐income countries. The search strategy was restricted to publications in English, which may have led to the exclusion of relevant studies in other languages. There are various terms used to describe decision‐making, acute care and prehospital care. Therefore, despite our efforts to develop a comprehensive search strategy, we may have missed some relevant articles. Grey literature was not included, which may have led to missing some additional results; however, the decision to exclude it was made to prioritise the inclusion of peer‐reviewed literature and ensure the quality of the data reviewed. During the complementary search, a limitation was identified when articles written by the research group were found. To avoid bias, none of these authors were involved in analysing the present findings. Finally, another limitation is that the protocol was not registered prior to publication. The reason for this is that the literature used in the review only suggests, but does not require, registration. In hindsight, registering the protocol would have strengthened the rigour and credibility of the study.

## 6 Conclusions

Shared decision‐making with older patients in acute prehospital settings is conditional, which implies decisions made by healthcare professionals. There is significant room for improvement in how healthcare professionals systematically approach this decision‐making process. Our findings show that decision‐making can vary depending on where it takes place, how it is conducted, and who is involved in the care of the older patient.

A process must take place to determine the older patient's decision‐making capacity and the perspectives of family members, and to gather information from anyone who knows the patient well, absent hierarchical barriers. This information must then be integrated with medical considerations. The approach should be driven by a genuine willingness to empathetically understand how patients’ life experiences influence their decisions. Considering these findings, we suggest that promoting shared decision‐making with older patients requires acknowledging the described conditional issues in both educational and clinical settings.

## Author Contributions

**Anders Sterner:** conceptualization, methodology, investigation, formal analysis, writing the original draft, writing – review and editing. **Bodil Holmberg:** investigation, validating, writing the original draft, writing – review and editing. **Anders Bremer:** conceptualization, methodology, writing the original draft, writing – review and editing. **Anders Svensson:** investigation, writing the original draft, writing – review and editing. **Henrik Andersson:** investigation, writing the original draft, writing – review and editing. **Catharina Frank:** investigation, formal analysis, writing the original draft, writing – review and editing.

## Ethics Statement

The authors have nothing to report.

## Consent

The authors have nothing to report.

## Conflicts of Interest

The authors declare no conflicts of interest.

## Supporting information


**Appendix S1:** Supporting Information.

## Data Availability

Data sharing not applicable to this article as no datasets were generated or analysed during the current study.
