# Supplementary material for: Decision‐Making for Older Patients in Acute Prehospital Situations: A Scoping Review
Source: Scand J Caring Sci. 2025 Oct 30;39(4):e70148. doi: 10.1111/scs.70148 (PMC12575412; doi:10.1111/scs.70148)
Supplement: Supplementary file 1 — Appendix S1: Supporting Information. [file SCS-39-0-s001.docx]

Supplementary file1. Comprehensive search strategies

PubMed.

| (("decision making, shared"[MeSH Terms] OR "Decision Making"[MeSH Terms] OR "shared decision making"[Title/Abstract] OR "Decision Making"[Title/Abstract]) AND ("Emergency Medical Services"[MeSH Terms] OR "Ambulances"[MeSH Terms] OR "prehospital"[Title/Abstract] OR "pre hospital"[Title/Abstract] OR "Ambulance*"[Title/Abstract])) AND ("Aged"[MeSH Terms] OR "Elderly"[Title/Abstract] OR "Senior*"[Title/Abstract] OR "Older"[Title/Abstract] OR "Geriatric"[Title/Abstract])  Filters: English, from 2000 - 2020 |
| --- |

Cinahl

| MH "Prehospital Care" OR MH "Emergency Medical Services" OR MH "Ambulances" OR ( prehospital OR "pre hospital" OR ambulance )  (MH "Decision Making+") OR ( MH "Decision Making, Shared" OR "decision making" or "shared decision making" )  (MH "Aged+") OR ( "Elderly" OR "Senior" OR "Older" OR "Geriatric" )  Limiters: Published Date: 20000101-20221231; English Language; Peer Reviewd |
| --- |

Scopus

| ( TITLE-ABS-KEY ( "Decision Making"  OR  "shared decision making" )  AND  TITLE-ABS-KEY ( "Emergency Medical Service*"  OR  prehospital  OR  "pre hospital"  OR  ambulance* )  AND  TITLE-ABS-KEY ( aged  OR  elderly  OR  senior*  OR  older  OR  geriatric ) )  AND  ( LIMIT-TO ( PUBYEAR ,  2022 )  OR  LIMIT-TO ( PUBYEAR ,  2021 )  OR  LIMIT-TO ( PUBYEAR ,  2020 )  OR  LIMIT-TO ( PUBYEAR ,  2019 )  OR  LIMIT-TO ( PUBYEAR ,  2018 )  OR  LIMIT-TO ( PUBYEAR ,  2017 )  OR  LIMIT-TO ( PUBYEAR ,  2016 )  OR  LIMIT-TO ( PUBYEAR ,  2015 )  OR  LIMIT-TO ( PUBYEAR ,  2014 )  OR  LIMIT-TO ( PUBYEAR ,  2013 )  OR  LIMIT-TO ( PUBYEAR ,  2012 )  OR  LIMIT-TO ( PUBYEAR ,  2011 )  OR  LIMIT-TO ( PUBYEAR ,  2010 )  OR  LIMIT-TO ( PUBYEAR ,  2009 )  OR  LIMIT-TO ( PUBYEAR ,  2008 )  OR  LIMIT-TO ( PUBYEAR ,  2007 )  OR  LIMIT-TO ( PUBYEAR ,  2006 )  OR  LIMIT-TO ( PUBYEAR ,  2005 )  OR  LIMIT-TO ( PUBYEAR ,  2004 )  OR  LIMIT-TO ( PUBYEAR ,  2003 )  OR  LIMIT-TO ( PUBYEAR ,  2002 )  OR  LIMIT-TO ( PUBYEAR ,  2001 )  OR  LIMIT-TO ( PUBYEAR ,  2000 ) )  AND  ( LIMIT-TO ( LANGUAGE ,  "English" ) ) |
| --- |

PsycInfo

| (MAINSUBJECT.EXACT.EXPLODE("Decision Making") OR noft("shared decision making" OR "Decision Making")) AND noft("Emergency Medical Services" OR prehospital OR "pre hospital" OR ambulance*) AND noft(Aged OR Elderly OR Senior* OR Older OR Geriatric) |
| --- |

Web of Science

| “Decision Making” OR “shared decision making” (topic) AND “Emergency Medical Service*” OR prehospital OR “pre hospital” OR “Ambulance*” (topic) AND aged OR elderly OR senior* OR older OR geriatric (topic) |
| --- |
